# Type I interferon limits interleukin-6 signalling in SLE through shedding interleukin-6 receptors

**DOI:** 10.1093/rheumatology/keaf281

**Published:** 2025-07-09

**Authors:** Martyna Hempel, Erik Klapproth, Annika Krause, Christoph Becker-Pauly, Sebastian Zeissig, Babett Heschel, Nadine Weser, Julia Fantana, Nicolai Leuchten, Stefan Rose-John, Ali El-Armouche, Adelheid Korb-Pap, Martin Aringer

**Affiliations:** Division of Rheumatology, Department of Medicine III, and interdisciplinary University Center for Autoimmune and Rheumatic Entities (UCARE), University Medical Center and Faculty of Medicine Carl Gustav Carus at the TU Dresden, Dresden, Germany; Department of Pharmacology, Faculty of Medicine Carl Gustav Carus at the TU Dresden, Dresden, Germany; Institute of Musculoskeletal Medicine, University Hospital Münster, Münster, Germany; Institute of Biochemistry, University of Kiel, Kiel, Germany; Department of Medicine I, and interdisciplinary University Center for Autoimmune and Rheumatic Entities (UCARE), University Medical Center and Faculty of Medicine Carl Gustav Carus at the TU Dresden, Dresden, Germany; Division of Rheumatology, Department of Medicine III, and interdisciplinary University Center for Autoimmune and Rheumatic Entities (UCARE), University Medical Center and Faculty of Medicine Carl Gustav Carus at the TU Dresden, Dresden, Germany; Department of Pharmacology, Faculty of Medicine Carl Gustav Carus at the TU Dresden, Dresden, Germany; Division of Rheumatology, Department of Medicine III, and interdisciplinary University Center for Autoimmune and Rheumatic Entities (UCARE), University Medical Center and Faculty of Medicine Carl Gustav Carus at the TU Dresden, Dresden, Germany; Division of Rheumatology, Department of Medicine III, and interdisciplinary University Center for Autoimmune and Rheumatic Entities (UCARE), University Medical Center and Faculty of Medicine Carl Gustav Carus at the TU Dresden, Dresden, Germany; Department of Medicine I, and interdisciplinary University Center for Autoimmune and Rheumatic Entities (UCARE), University Medical Center and Faculty of Medicine Carl Gustav Carus at the TU Dresden, Dresden, Germany; Department of Pharmacology, Faculty of Medicine Carl Gustav Carus at the TU Dresden, Dresden, Germany; Institute of Musculoskeletal Medicine, University Hospital Münster, Münster, Germany; Division of Rheumatology, Department of Medicine III, and interdisciplinary University Center for Autoimmune and Rheumatic Entities (UCARE), University Medical Center and Faculty of Medicine Carl Gustav Carus at the TU Dresden, Dresden, Germany

**Keywords:** lupus, disease activity, interleukin-6, type I interferon, receptor shedding, trans-signalling

## Abstract

**Objective:**

To fully understand why C-reactive protein (CRP) is usually only mildly elevated in active systemic lupus erythematosus (SLE), although interleukin-6 (IL-6) is increased, but is high in SLE patients with bacterial infections.

**Methods:**

Sera and peripheral blood mononuclear cells (PBMCs) of SLE patients and healthy individuals were investigated. IL-6 and soluble IL-6 receptor (sIL-6R) were measured by ELISA. Membrane IL-6 receptor-α (CD126), gp130 (CD130) and signal transducer and activator of transcription 3 (STAT3) phosphorylation after IL-6 stimulation were analysed by flow cytometry. PBMCs and HepG2 liver cells were stimulated with various cytokines, and IL-6 receptor shedding was determined by immunoprecipitation and western blotting of supernatants. HEK-293 T cells were transfected with wild type CD126 or a shedding-resistant mutant, and soluble sIL-6R was measured following cytokine stimulation.

**Results:**

While sIL-6R and IL-6 were increased with SLE activity, CD126 positive lymphocytes were decreased in SLE. IL-6 plus IFNα decreased CD126 on lymphocytes, but increased sIL-6R in their supernatant, detected by immunoprecipitation and western blotting. IL-6 plus IFNα on HepG2 liver cells resulted in a similar sIL-6R increase. Increased supernatant sIL-6R was seen in HEK-293 T cells transfected with wild type CD126, but not those transfected with the shedding-resistant mutant. STAT3 phosphorylation upon IL-6 stimulation was reduced.

**Conclusion:**

The combination of IL-6 and type I IFN induces shedding of CD126 to sIL-6R, shifting IL-6 signalling to trans-signalling. In situations of IL-6 excess over sIL-6R only, IL-6 or IL-6–sIL-6R complexes reach the liver and increase CRP. These findings not only explain the discrepancy in SLE, but also have implications for severe viral infection.

Rheumatology key messages

- CRP is high in infections in SLE, but not in active SLE, despite increased IL-6.
- Type I interferon and IL-6 together lead to leukocyte and hepatocyte IL-6 receptor shedding.
- The resulting soluble IL-6 receptor/gp130 buffer prevents CRP production unless IL-6 is very high.

## Introduction

CRP expression is directly induced by IL-6 [1, 2]. Accordingly, IL-6 and CRP correlate well under most circumstances. SLE is a notable exception to this rule. In SLE, CRP only occasionally shows a relevant increase. While increased CRP can be associated with lupus arthritis or serositis, high CRP usually is an indication of bacterial infection in SLE patients [3]. This also implies that CRP can increase to high levels in SLE patients, but is not usually associated with SLE disease activity.

In contrast, a body of literature reports increased IL-6 serum levels of patients with active SLE [4–7]. Thus, there is an apparent discrepancy between IL-6 and CRP levels in this disease. In particular, higher CRP levels cannot be found in many situations where IL-6 levels are clearly elevated. An association between IL-6 and CRP was only found by some authors, and usually on a level lower than expected [6, 8–14].

One finding that could have explained some of this effect is the occurrence of antibodies against CRP [15]. However, while these antibodies may have some effect, the lack of association with CRP speaks against this hypothesis [16]. In addition, their prevalence would be too low to fully explain the low CRP levels in SLE, and these antibodies would not explain the fact that severe bacterial infections, in the same patients, still lead to high CRP levels.

More relevant are data linking the apparent disconnect between IL-6 and CRP to both CRP gene variants and the influence of IFNα [17, 18]. While polymorphisms reducing CRP expression were found in less than half of the SLE patients, and are therefore only relevant for the degree of correlation, an influence of type I IFN seemed a more plausible explanation. This would also fit the pattern that viral infections, which usually lead to type I IFN production, are associated with lower CRP levels than bacterial infections [19].

On a mechanistic level, the underlying mechanisms have not yet been elucidated. We therefore investigated IL-6, IL-6 receptors and IL-6 signal transduction to shed light on this question. In leukocytes and hepatocytes, IL-6 transmits its signals via a β-receptor consisting of two chains of gp130 (CD130), a common receptor of several cytokines, and one chain of its cognate IL-6 receptor α (CD126). The assembly of this complex leads to activation of Janus kinases (JAKs) constitutively bound to the cytoplasmic portion of gp130 and subsequent tyrosine phosphorylation and activation of signal transducer and activator of transcription 3 (STAT3) [20].

Importantly, IL-6 is also able to signal into cells that do not contain CD126 on their surface [21]. Whereas CD126 expression is limited to leukocytes, hepatocytes and intestinal epithelium [22, 23], essentially all cells express the signalling receptor gp130, which binds JAK1 and STAT3 and transmits the intracellular signal. In the absence of surface CD126, cells assemble the signalling complex via soluble IL-6 receptors bound to IL-6, which consecutively bind gp130 on cells in a process termed trans-signalling [24–26].

The soluble receptors capable of trans-signalling derive from membrane-bound IL-6 receptors shed by ADAM family metalloproteinases [27, 28]. Soluble gp130 (sgp130), generated by alternative splicing and shedding, forms a functional IL-6 buffer together with soluble IL-6 receptor (sIL-6R) [21, 29]. Accordingly, our investigations focused on both surface and soluble IL-6 receptors, as well as IL-6 receptor signalling, to survey the modifications in SLE.

## Methods

### Patients

Peripheral venous blood was obtained from 41 patients fulfilling EULAR/ACR criteria for SLE [30] (85% female, median age 40 [range 21–79] years), 33 patients with rheumatoid arthritis (RA) fulfilling EULAR/ACR criteria [31] (76% female, 54 [32–87] years), and 71 healthy individuals (68% female, 34 [21–84] years). All patients and healthy individuals gave their informed consent, and the local ethics committee approved the study. SLE disease activity was evaluated by European Consensus Lupus Activity Measure (ECLAM) [32]. Routine laboratory values were taken from the patients’ charts. Patients with clinically suspected infections were excluded.

### ELISAs

Serum was prepared after clotting in anticoagulant-free tubes (Sarstedt, Nümbrecht, Germany). Levels of IL-6 and sIL-6R were measured by ELISA (R&D Systems, Minneapolis, MN, USA).

### Peripheral blood mononuclear cells and cell staining

Peripheral blood mononuclear cells (PBMCs) were prepared from fresh venous blood anticoagulated with heparin (Lithium-Hep, Sarstedt, Germany), using Leucosep tubes (Greiner bio-one, Kremsmünster, Austria) and Bicoll separation medium (Biochrom AG, Berlin, Germany). CD126 and CD130 were stained with phycoerythrin (PE)-labelled specific or control antibodies (BD Biosciences, San Jose, CA, USA). For determining tyrosine-phosphorylated STAT3 (pSTAT3), PBMCs were left unstimulated or stimulated with recombinant human IL-6 (100 ng/ml) (Dianova, Hamburg, Germany) for 10 and 15 min, fixed with formaldehyde (2%), permeabilized with methanol (80%) and stained with PE-labelled anti-pSTAT3 or control antibodies (BD Biosciences). Cells were analysed on a BD FACSCalibur flow cytometer, gating for lymphocytes. Mean fluorescence intensity (MFI) was used as a semiquantitative measure of the pSTAT3 contents.

### PBMC 24 h stimulations

PBMCs were resuspended in RPMI 1640 plus l-glutamine (Thermo Fisher Scientific, Paisley UK), penicillin, streptomycin and 10% heat-inactivated fetal calf serum (PAA Laboratories, Pasching, Austria) at 1 × 10^6^ cells per mL. The following cytokines were added to the cell suspension: IL-6 (250 ng/ml) (Dianova, Hamburg, Germany), TNFα (50 ng/ml), IFNγ (100 ng/ml), IL-10 (100 ng/ml), IFNα (1 U/ml) (all R&D Systems), or IL-21 (50 ng/ml) (PeproTech, Princeton, NJ, USA), separately and in combinations. Cells were incubated for 24 h at 37°C, 5% CO_2_, and washed twice with phosphate-buffered saline (Sigma-Aldrich, Taufkirchen, Germany).

### Stimulation of the human liver cell line HepG2

HepG2 cells (ATCC, HB-8065) were cultured in Eagle’s minimum essential medium (EMEM) (ATCC, 30-2003) plus penicillin and streptomycin with 10% heat-inactivated fetal calf serum (PAA Laboratories) on six-well plates. After confluence reached about 60%, phorbol myristate acetate (PMA, Sigma-Aldrich) at a concentration of 25 or 50 ng/ml or the following cytokines were added to the wells: IL-6 (250 ng/ml) (Dianova, Hamburg, Germany), TNFα (50 ng/ml), IFNα (1 U/ml) (R&D Systems), separately and in combinations. Cells were incubated for 24 h at 37°C, 5% CO_2_. Cell suspensions were centrifuged and supernatant was collected for immunoprecipitation and western blotting.

### Immunoprecipitation of soluble IL-6R

Supernatants of stimulated PBMCs were centrifuged, and total soluble protein was measured by BCA assay (Thermo Fisher Scientific, Waltham, MA, USA). Supernatants were pre-cleared using 50 μl of a protein AG sepharose slurry (50% v/v) (Alpha Diagnostics, Reinach, Switzerland). Primary antibodies (CD126, 551462, BD Biosciences) were added to the 0.75 mg protein lysate and rotated for 1 h at 4°C. Subsequently, 50 μl of a protein AG sepharose slurry (50% v/v) was added and rotated overnight at 4°C. Immunoprecipitates were washed three times with cold lysis buffer. Precipitated proteins were boiled in 50 μl sample buffer, separated by SDS–PAGE, transferred, and blotted with an anti-IL-6R antibody (ab128008, Abcam, Cambridge, UK) and horseradish peroxidase-conjugated donkey anti-rabbit antibodies (Sigma-Aldrich). Proteins were visualized using ECL substrate (SuperSignal™ West Dura Extended Duration Substrate, Thermo Fisher Scientific) and images were acquired using a Fusion FX chemiluminescence imaging system (Vilber, Marne-la-Vallée, France). Densitometric analysis was performed using the FusionCapt Advance software (Vilber).

### HEK293T cell transfection with wild type and mutant IL-6R

HEK293T (ATCC, Manassas, VA, USA) cells were cultured in DMEM High Glucose (Sigma-Aldrich) containing 1% Pen/Strep (Life Technologies, Darmstadt, Germany) and 10% heat inactivated FCS (Sigma-Aldrich). For transfection studies, 10^5^ cells were seeded in 6 well plates, transfected using jetPRIME DNA transfection reagent (Polyplus, Illkirch, France) and previously described IL-6R variants [33]. FACS analyses were conducted to assess transfection efficiency using PE mouse anti-human CD126 (BD Pharmingen, Heidelberg, Germany) and PE mouse IgG1 Isotype Control (BD Pharmingen) on a FACS Canto flow cytometer (BD Biosciences, Heidelberg, Germany). After 24 h of transfection, cells were incubated with recombinant hIL-6 (250 ng/ml) (Dianova, Hamburg, Germany) and IFN type I (100 ng/ml) (PBL Assay Science, Piscataway, NJ, USA). Following an additional 24-h period, supernatant was harvested and the soluble receptor was directly precipitated [34]. Cell lysates were prepared using RIPA buffer and cOmplete™ EDTA-free Protease Inhibitor Cocktail (Sigma-Aldrich). Western blot analysis was performed using a mouse anti-human IL-6R (4–11) as primary antibody [33], and glyceraldehyde 3-phosphate dehydrogenase (GAPDH) (CST, Frankfurt, Germany) was used as loading control.

### Statistical analysis

Student’s *t*-test (with Welch’s corrections when variations were different) and the Mann–Whitney test were used for group comparisons in the setting of normally and non-normally distributed groups. For testing associations, Pearson’s and Spearman’s correlation coefficients were calculated, respectively. *P*-values <0.05 were considered statistically significant. Statistics with fewer than seven data points were considered nominal. While *P*-values in the *in vitro* experiments focus on one result, multiple comparisons (*n* = 18) were performed between the patient-derived data. If Bonferroni-corrected, this would demonstrate significance only for *P*-values <0.0027.

## Results

### Treatment and active organ involvement of SLE

Of the 41 SLE patients, 37 (90%) were on hydroxychloroquine and all but one (98%) on glucocorticoids with a median dose of 5 mg q.d. (range 2–40 mg q.d.). In addition, 14 patients (34%) were treated with azathioprine, six (15%) with methotrexate and four (10%) with mycophenolate. On the day when blood was drawn, eight patients (20%) had active arthritis, three (7%) serositis, and two (5%) active lupus nephritis. Seven patients (17%) had a secondary anti-phospholipid syndrome and were anticoagulated. The median ECLAM was 2 (range 0–6).

### Serum IL-6

**Figure 1.**
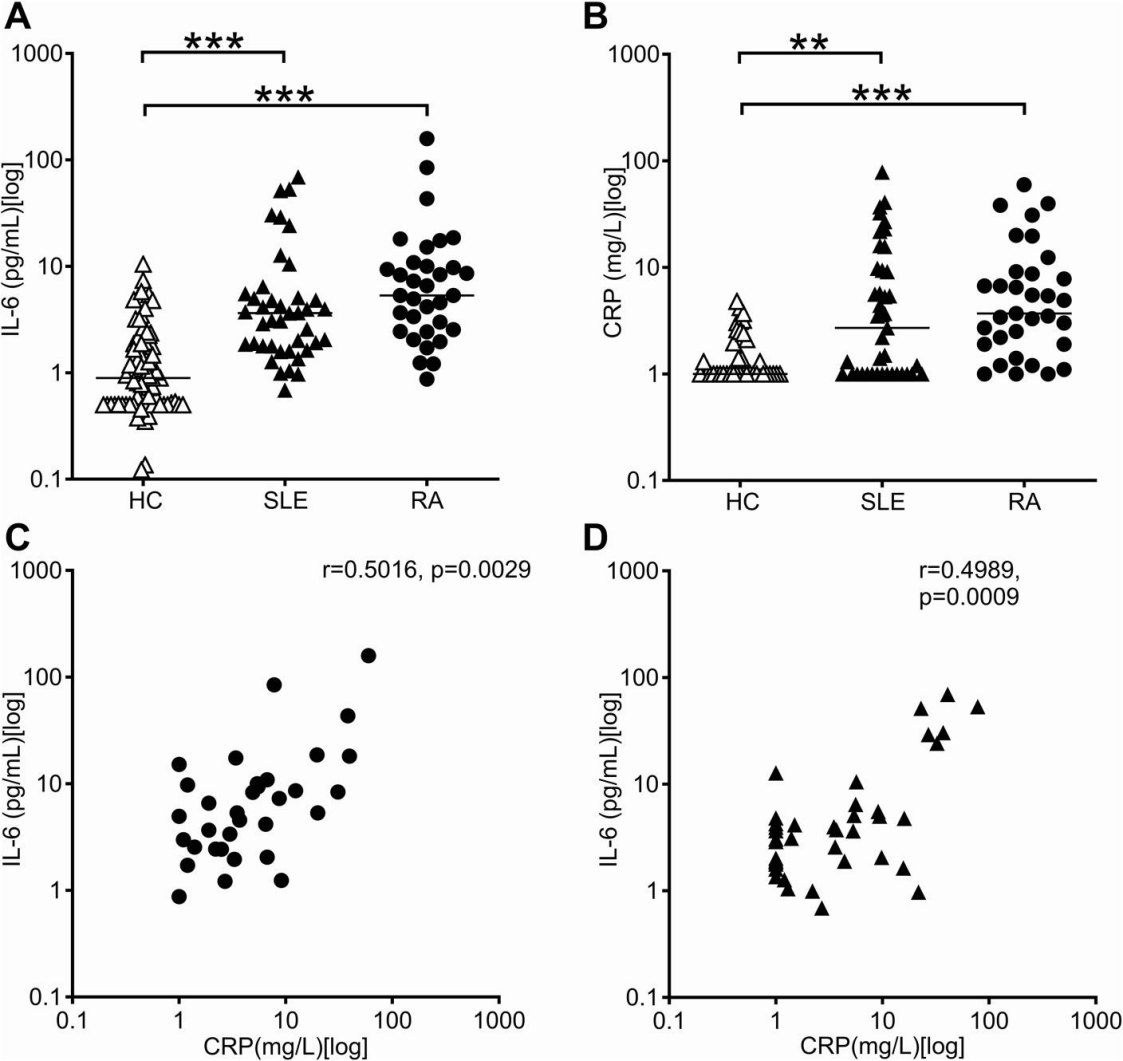
Serum IL-6 and its correlation with CRP. IL-6 was measured by ELISA in sera of 71 healthy individuals (HC), 41 patients with SLE and 33 patients with rheumatoid arthritis (RA). (**A**) IL-6 levels were significantly increased in both patients with SLE (filled triangles) and patients with RA (filled circles). (**B**) CRP levels were also significantly increased in both patients with SLE (filled triangles) and patients with RA (filled circles). Note the logarithmic *y*-axes. (**C**) In RA sera, IL-6 was correlated with CRP, as expected. (**D**) IL-6 correlated with CRP also in SLE sera, where this was mostly due to six sera with very high IL-6 levels. **P < 0.01, ***P < 0.001

In accordance with the literature, we found that a considerable proportion of the SLE patients had increased IL-6 levels in their sera (Fig. 1A), and that IL-6 was significantly elevated in SLE (median 3.64 [range 0.69–69.26] *vs* 0.89 [0.12–10.49] pg/ml in healthy individuals, *P < *0.0001) and correlated with SLE disease activity measured by ECLAM (Spearman *r* = 0.40, *P = *0.0088) (Supplementary Fig. S1A, available at *Rheumatology* online). The 11 patients with active serositis or arthritis had significantly (*P < *0.0001) higher serum IL-6 levels (median 24.21 [3.64–69.26] pg/ml) than those without (median 2.033 [0.6885–6.450] pg/ml). When we compared the SLE serum data with those of RA patients, the latter had a tendency towards even higher levels of IL-6 (5.33 [0.87–158.50] pg/ml, *P = *0.06 *vs* SLE) (Fig. 1A).

### IL-6 and CRP

CRP levels were increased in SLE (*P = *0.0018), but more commonly (*P < *0.0001) increased in RA (Fig. 1B), even though the difference between SLE and RA was not significant (*P = *0.1780). IL-6 correlated with CRP in SLE patients (*r* = 0.50, *P = *0.0009) (Fig. 1C). In contrast to IL-6, however, CRP was not significantly correlated (*r* = 0.30, *P = *0.0624) with SLE disease activity (Supplementary Fig. S1B, available at *Rheumatology* online). In RA, IL-6 likewise correlated with CRP (*r* = 0.50, *P = *0.0029) (Fig. 1C). While the RA distribution appeared more continuous (Fig. 1C), the distribution for SLE was reminiscent of two distinct populations (Fig. 1D). This led us to exploratory testing of the correlation in patients with low level IL-6 only. When including only IL-6 levels of 20 pg/ml or lower, the correlation for SLE was lost (*r* = 0.18, *P = *0.29), whereas the correlation for RA was maintained (*r* = 0.38, *P = *0.04) (Supplementary Fig. S2, available at *Rheumatology* online). These data are consistent with the idea that IL-6 is increased in SLE, but not directly associated with increased CRP, unless IL-6 levels are high.

### Soluble IL-6 receptor levels

**Figure 2.**
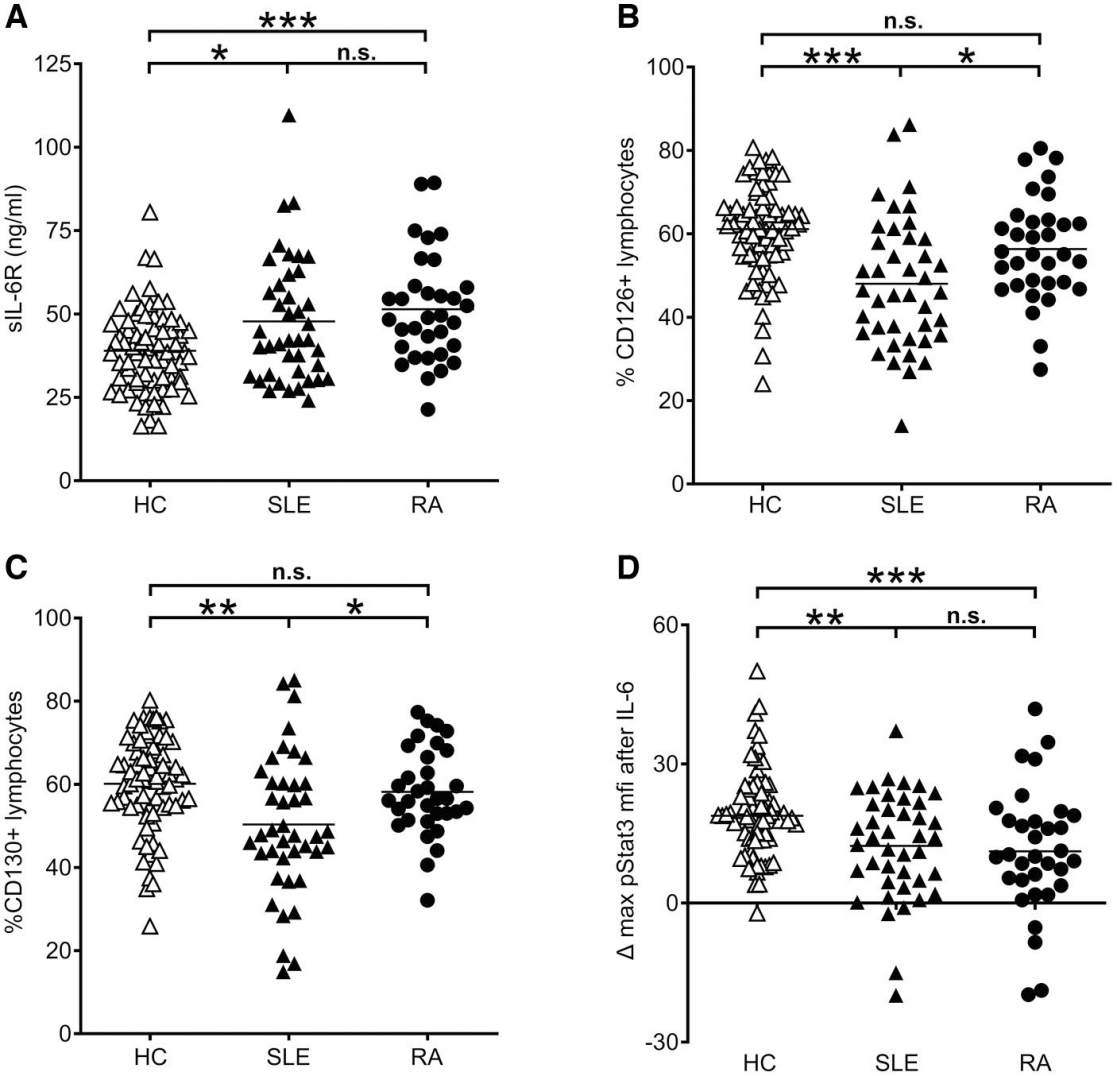
Soluble and membrane-bound IL-6 receptor in SLE and RA, and IL-6 signalling. (**A**) Serum soluble IL-6 receptor measured by ELISA was increased in both SLE (*P = *0.02) and RA (*P = *0.0001). The difference between SLE and RA was not significant (*P = *0.1931). The percentages of lymphocytes carrying surface IL-6 receptor-α (CD126) and gp130 (CD130) was determined by direct immunofluorescence and flow cytometry. (**B**) CD126 was significantly decreased in SLE (filled triangles) as compared with healthy individuals (HC, open triangles, *P < *0.0001), but not in RA patients (filled circles, *P = *0.0579 *vs* HC, *P = *0.0149 *vs* SLE). (**C**) The percentage of CD130 positive lymphocytes was also reduced in SLE, but to a lesser degree (*P = *0.0015), and not reduced in RA patients (*P = *0.4012 *vs* HC, *P = *0.0168 *vs* SLE. (**D**) Peripheral blood mononuclear cells (PBMCs) were stimulated with recombinant IL-6 for 10 and 15 min, and the mean fluorescence intensity (MFI) of phosphorylated signal transducer and activator of transcription 3 (STAT3) was measured by direct intracellular immunofluorescence and flow cytometry. The maximum increase in pSTAT3 (at either 10 or 15 min) was less pronounced in SLE than in HC (*P = *0.0044) and similarly reduced in RA (*P = *0.0006 *vs* HC, *P = *0.5739 *vs* SLE). *P < 0.05; **P < 0.01, ***P < 0.001

Since sIL-6R can switch IL-6 signals to trans-signalling, we investigated sIL-6R in SLE sera (Fig. 2A). Indeed, sIL-6R levels were increased as compared with healthy individuals (median 42.2 [24.1–109.6] *vs* 38.6 [16.3–80.5] ng/ml, *P = *0.02). This was also seen for RA (mean [s.d.] 51.4 [16.1] ng/ml; median 48.9 ng/ml; Mann–Whitney *P = *0.0001).

### Surface IL-6 receptor expression

Since IL-6R mostly derives from shedding of membrane IL-6R of either hepatocytes or leukocytes, we at the same time investigated IL-6 receptor surface expression on leukocytes, given their easier accessibility. In contrast to the increased levels of sIL-6R, the percentage of lymphocytes carrying the IL-6 receptor α-chain was significantly lower in SLE (mean [s.d.] 48 [16]%; median 46 [14–86]%; Fig. 2B) than in healthy individuals (median 61 [24–81]%; Mann–Whitney *P < *0.0001). RA patients showed a trend towards decreased CD126^+^ lymphocytes (mean [s.d.] 52 [11]%; median 55 [27–81]%; Mann–Whitney *P = *0.0579) compared with healthy individuals, but higher (*P = *0.0149) than SLE patients.

CD130^+^ lymphocytes were slightly decreased in SLE (50 [17] *vs* 60 [11]% in healthy individuals, *P = *0.0015), while the CD130^+^ lymphocytes of RA patients (58 [10]%) were not different from those of healthy individuals (Fig. 2C). In SLE, percentages of CD126^+^ and CD130^+^ lymphocytes correlated (*r* = 0.82, *P < *0.0001).

### Negative correlation membrane and soluble IL-6-receptor

There was a significant negative correlation between (diminished) surface and (increased) soluble IL-6 receptor α (Spearman *r* = −0.36, *P = *0.022) (Supplementary Fig. S3, available at *Rheumatology* online). Thus, the findings of decreased CD126, increased sIL-6R and their significant negative correlation were consistent with the hypothesis that SLE involved increased IL-6 receptor shedding. On an exploratory basis, we formed a ratio between the percentage of CD126^+^ lymphocytes and the corresponding sIL-6R level. This ratio was (*P < *0.0001) diminished in SLE as compared with healthy individuals, with a median of 0.95 [0.26–2.64] and 1.48 [0.58–4.74], respectively (Supplementary Fig. S4A, available at *Rheumatology* online). The CD126/sIL-6R ratio correlated (*r* = 0.32, *P = *0.041) with a ratio of CRP/IL-6 (Supplementary Fig. S4B, available at *Rheumatology* online). Accordingly, the shift towards soluble IL-6R correlated with low CRP. Serum sIL-6 receptor alone, in fact, correlated even slightly better (Spearman *r* = −0.36, *P = *0.0209) with the CRP/IL-6 ratio.

### Functional consequences of diminished IL-6 receptors

When we stimulated leukocytes of SLE patients with recombinant human IL-6, lymphocytes of SLE patients had a diminished increase in STAT3 phosphorylation compared with healthy individuals (Fig. 2D). The maximum increase in the pSTAT3 MFI, reached after either 10 or 15 min, amounted to 12.4 [11.5] (median 14.2) for SLE, whereas it reached a median of 18.8 [−2.2 to 50.1] in healthy individuals’ lymphocytes (Mann–Whitney *P = *0.0044). Thus, the diminished IL-6 receptor expression on SLE lymphocytes appeared to hamper the ability of these lymphocytes to respond to IL-6. Similar results were obtained with RA lymphocytes (Fig. 2D).

### Lymphocyte stimulation with cytokine mimics the ex vivo situation

**Figure 3.**
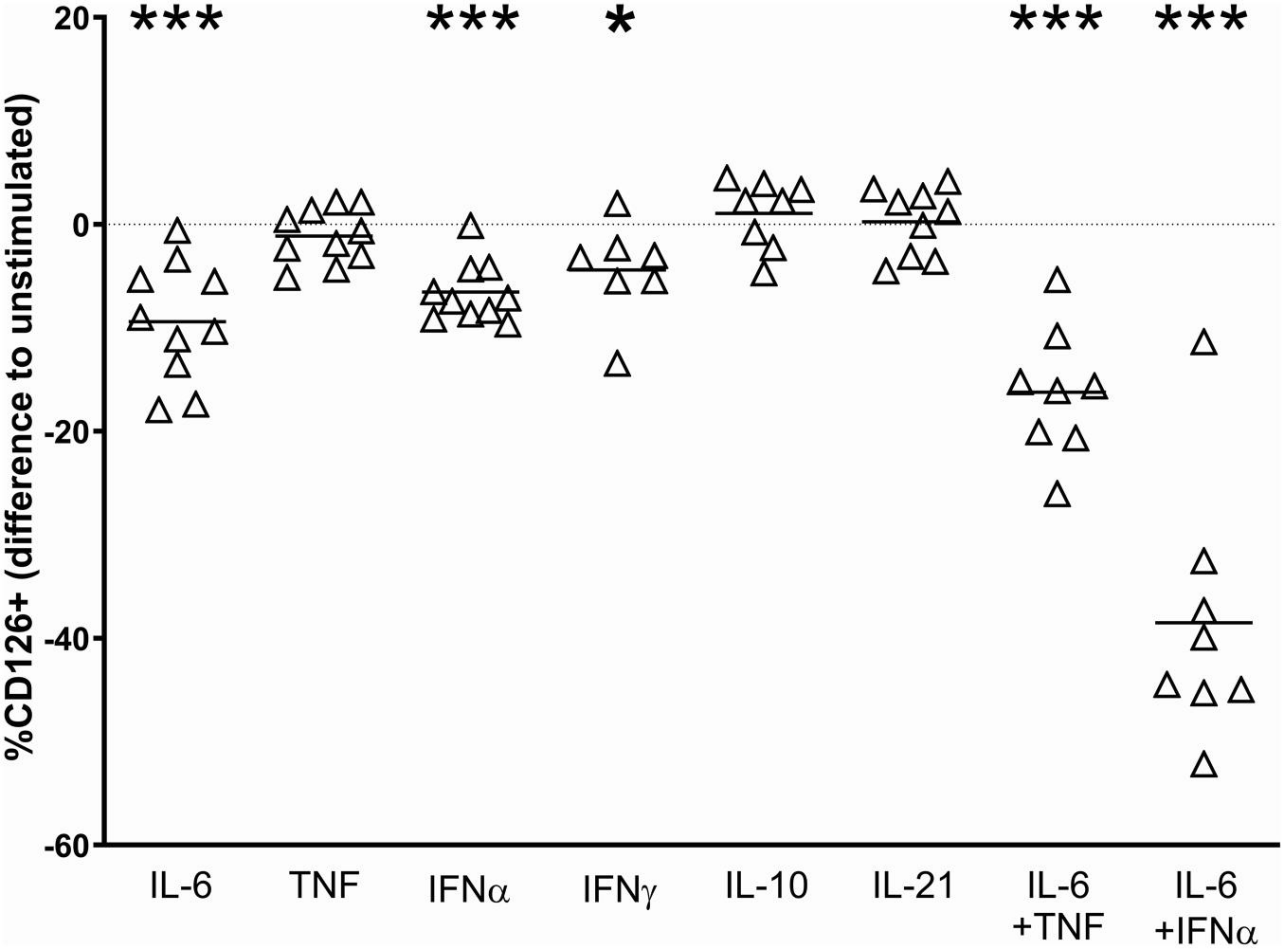
CD126 decrease and supernatant sIL-6R increase induced by IL-6 and IFNα. Peripheral blood mononuclear cells of eight healthy individuals were left unstimulated or stimulated with TNF, IL-6, IL-10 IL-21, IFNα, IFNγ, or combinations of these cytokines, for 24 h *in vitro*. Lymphocyte CD126 was measured by direct fluorescence and flow cytometry. Significant decreases in the percentage of CD126 positive lymphocytes were seen with IL-6 (*P = *0.0006), IFNα (*P < *0.0001), a minimal decrease with IFNγ (*P = *0.047). The combination of IL-6 and IFNα induced the strongest decrease (*P < *0.0001), but also the combination of IL-6 and TNF had more effect than single cytokines (*P = *0.0002). *P < 0.05; **P < 0.01, ***P < 0.001

Since IL-6 receptor shedding by leukocytes appeared important for the question of inadequately low CRP, we next investigated whether we could induce a similar reduction in CD126 by cytokines increased in SLE. As shown in Fig. 3, 24 h of stimulation with TNF, IL-10 and IL-21 did not influence, and IFNγ only minimally (−4.4 [4.7]%, *P = *0.047) influenced, CD126 surface expression. IL-6 and IFNα had a very modest effect, reducing the expression by 9.4 [5.8]% (*P = *0.0006) and 6.6 [2.9]% (*P < *0.0001), respectively. In combination, IL-6 and TNF induced a greater reduction (−16.2 [6.3]%; *P = *0.0002). However, IL-6 and IFNα together led to a CD126 decrease by 38.5 [12.5]% (*P < *0.0001), modelling the *ex vivo* situation in SLE patients.

### Increase in supernatant soluble IL-6R upon cytokine stimulation

**Figure 4.**
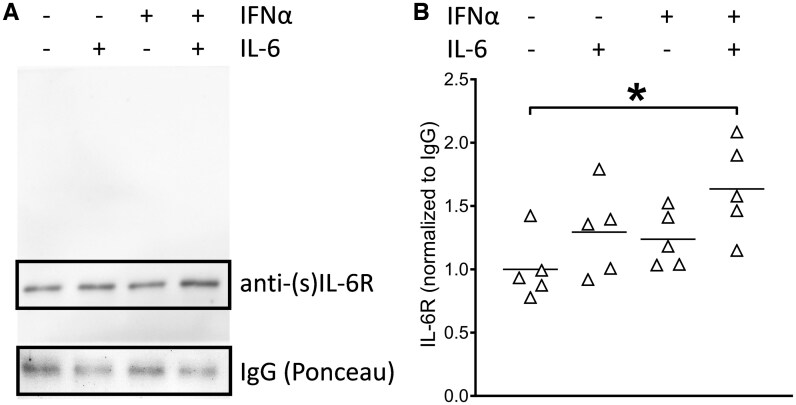
Increase in (shed) soluble IL-6 receptor after 24 h IL-6 plus IFNα stimulation of peripheral blood mononuclear cells (PBMCs). PBMCs of healthy individuals were left unstimulated or stimulated with IL-6, IFNα, or the combination of these cytokines for 24 h *in vitro*. Supernatants were tested by immunoprecipitation and western blotting against soluble IL-6 receptor (sIL-6R). (**A**) Western blot of one of four independent experiments. (**B**) Densitometric measurements of sIL-6R normalized to IgG for all five individual experiments, showing increased sIL-6R with the combination of both IL-6 and IFNα (nominal *P = *0.0055). *P < 0.05

The decrease in surface IL-6R was likely caused by IL-6R shedding. Unfortunately, several ELISA-(like) systems tested failed to reliably detect IL-6R once IL-6 was added. We therefore used immunoprecipitation of supernatants of healthy PBMCs to investigate the sIL-6R amounts present after 24 h of stimulation with IL-6, IFNα or the combination of both. As shown in Fig. 4, combined IFNα plus IL-6 led to an increase in detectable soluble IL-6R (Fig. 4A). Normalized for IgG, the nominal *P*-value across five experiments was significant (*P = *0.0055) (Fig. 4B).

### IL-6 Receptor is shed also from human hepatocytes

**Figure 5.**
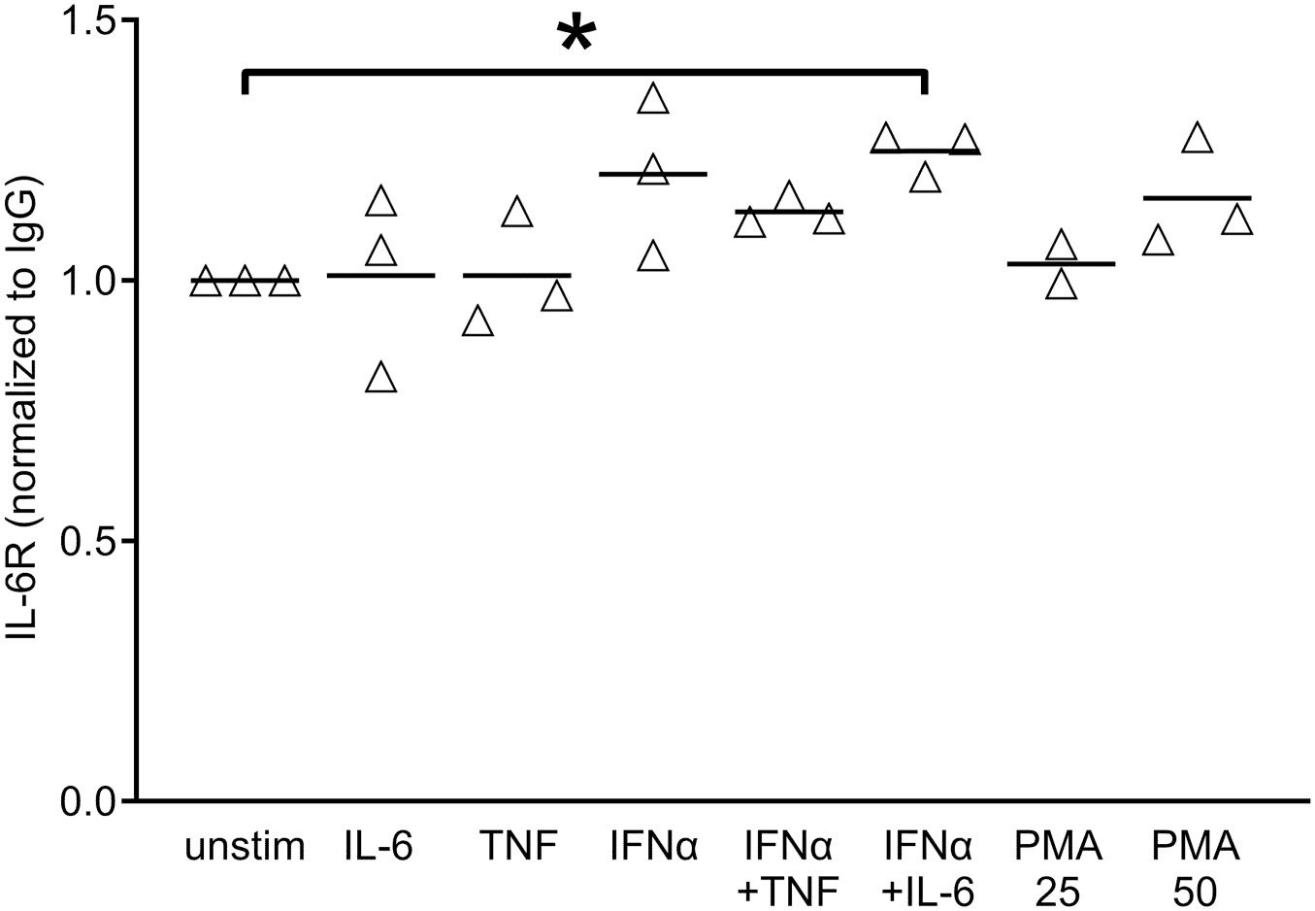
Increase in (shed) sIL-6R after 24 h stimulation of the HepG2 human hepatocyte cell line. HepG2 cells were left unstimulated or stimulated with IL-6, TNF, IFNα or combinations of these cytokines, or with phorbol myristate acetate (PMA; 25 and 50 ng/ml), for 24 h *in vitro*. Supernatants were tested by immunoprecipitation and western blotting against soluble IL-6 receptor (sIL-6R). Densitometric measurements of sIL-6R normalized to IgG for three individual experiments, showing increased sIL-6R with the combination of both IL-6 and IFNα (nominal *P = *0.0135). *P < 0.05

Like leukocytes, hepatocytes constitutionally express surface IL-6 receptors. We therefore explored whether shedding induced by IFNα plus IL-6 was also active in hepatocytes and could contribute to the generation of sIL-6R, testing the same cytokine combination on the human hepatocyte cell line HepG2, PMA as a positive control for shedding, and the same immunoprecipitation and western blotting approach for detecting shed IL-6R in supernatants. Indeed, the combination of IL-6 and IFNα led to increased shedding also of HepG2 human hepatocyte IL-6 receptor α-chains (Fig. 5).

### Increase of sIL-6R with wild type, but not shedding-resistant IL-6R

**Figure 6.**
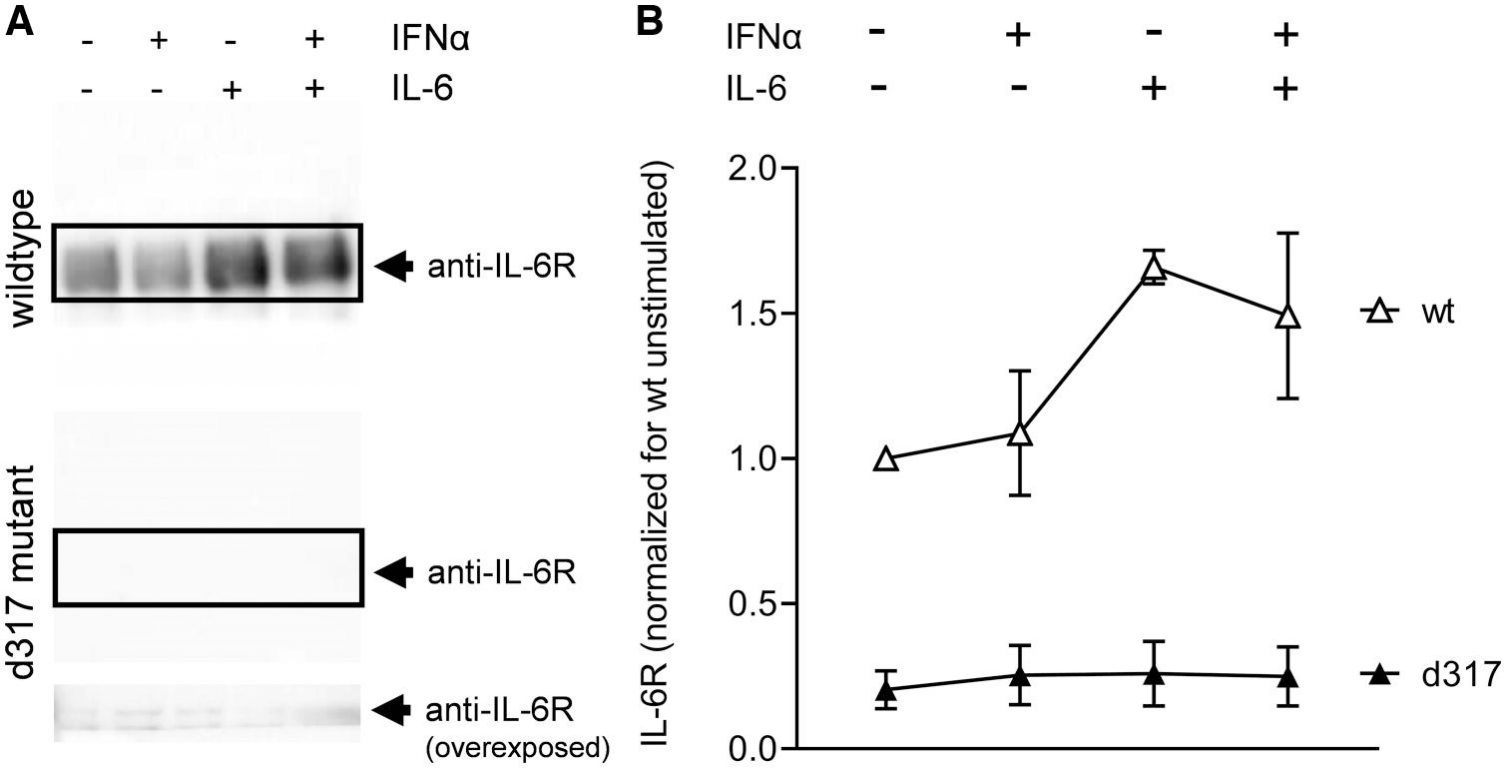
Increase in sIL-6R after stimulation blocked in shedding-resistant IL-6R mutant. HEK293T cells were transfected with either wild type (WT) IL-6R or the shedding resistant IL-6R d317 mutant (Δ317–352). The transfected HEK293T cells were left unstimulated or stimulated with IL-6, IFNα or the combination of these cytokines for 24 h *in vitro*. Supernatants were tested by immunoprecipitation and western blotting against soluble IL-6 receptor (sIL-6R). (**A**) Western blot of wild type (upper panel) and d317 (lower panel) supernatant. (**B**) Densitometric analysis normalized for supernatant of unstimulated HEK293T cells transfected with wild type IL-6R

IL-6 receptor shedding to sIL-6R is effected by metalloproteinases [33]. If the IL-6 and IFNα effect was due to shedding, one would expect to abrogate the effect with a metalloproteinase-resistant IL-6R construct. Accordingly, HEK293T cells were transfected either with wild type IL-6R or the shedding resistant IL-6R mutant Δ317-352 (d317) [33], with a transfection efficacy of >75%. The increase in sIL-6R in the supernatant of the HEK293T cells stimulated with IL-6 (and IFNα) was found when cells were transfected with wild type IL-6R, but was not present when cells were transfected with d317, where only minimal amounts of sIL-6R were detected in the supernatant (Fig. 6).

## Discussion

In this study, we provide, on a mechanistic level, the first full explanation for the well-described low CRP values in SLE and the discrepancy between IL-6 and CRP in the disease. We found that the combination of type I IFN and IL-6, both established as increased in SLE, leads to metalloproteinase-induced shedding of surface CD126 to sIL-6R. This shifts IL-6 signalling away from leukocytes and hepatocytes towards trans-signalling [25, 26]. In severe bacterial infection, the extremely high IL-6 levels overrun the so-formed IL-6 buffer, and CRP rises. These data transfer an important immunological concept to the SLE immune response and also refute an argument against a relevant role of IL-6 in SLE.

We have confirmed increased IL-6 in active SLE, which only partly translated into increased CRP, despite the fact that *CRP* is a directly IL-6 driven gene. We have also found a significant shift from membrane CD126 to sIL-6R. While not leading to total absence of surface receptors, the decrease in the membrane-bound receptor was obviously important enough to hamper IL-6 signalling: when stimulating cells with recombinant IL-6, we observed a clearly diminished increase in the phosphorylation of STAT3, the main signalling pathway of IL-6 [20]. This could not be directly tested for hepatocytes. However, a similar molecular situation had already been shown to have a comparable effect on hepatocytes [35].

We next searched for factors that could explain the shedding process in active SLE. We first explored the possibility that IL-6 itself would downmodulate IL-6 receptor expression. This indeed seemed to be the case, but only to a very modest degree. Other cytokines, and TNF in particular, appeared not to play a role in the regulation of IL-6 receptor expression. The only exception was some IFNα-induced IL-6 receptor loss. In contrast, combinations of IFNα and IL-6, or to a lesser degree, of TNF and IL-6, were identified as major regulators of IL-6 receptor expression.

Further experiments directly showed the shedding of IL-6R in this process. On the one hand, by immunoprecipitation, we detected an increase in sIL-6R in the supernatant of PBMCs stimulated with IFNα and IL-6. As expected, increased IL-6R shedding was also found from hepatocytes stimulated with both IL-6 and IFNα. On the other hand, the increase in sIL-6R in stimulated HEK293T cells was prevented when they were transfected with a mutant of the IL-6R instead of the wild type resistant to shedding by the metalloproteinases ADAM10 and ADAM17, whose activity is associated with IL-6 and IFNα [36, 37].

IFNα, IL-6 and TNF are increased in SLE [3, 6, 38–40]. They are produced in response to the immune complex deposition that characterizes the inflammatory organ manifestations of the disease. While TNF and IL-6 are mostly produced by monocytes and macrophages under these circumstances, IFNα mainly is a product of plasmacytoid dendritic cells [41], and IFNκ, another type I cytokine binding the same receptor, is produced by keratinocytes [42]. Thus, cytokines known to be released upon immune complex deposition led to the situation observed *in vivo* in SLE, and therefore provide for a meaningful explanation in the setting of this disease.

Our findings confirm, but expand on, the findings of Helena Enocsson and colleagues that IFNα inhibits IL-6 induced CRP transcription and secretion [17, 18, 43]. They also align well with clinical observations in SLE: in active SLE, IL-6 is usually increased, but only to a moderate level. At the same time, in combination with IFNα, IL-6 leads to the shedding of IL-6 receptors, from both leukocytes and hepatocytes. This will lead to the formation of IL-6–sIL-6R complexes.

In the presence of sgp130, such IL-6–sIL-6R complexes are blocked from direct trans-signalling, resulting in reduced hepatocyte IL-6R signalling as compared with moderate levels of IL-6 alone [35]. This was shown for patients with an IL-6R polymorphism that led to an approximately 2-fold increase in sIL-6R [35]. High levels of IL-6 still lead to hepatocyte IL-6R signalling, indicating that the sIL-6R/sgp130 buffer is then exhausted [21, 35]. This twofold increase in sIL-6R [35] is similar to the situation now found in SLE.

At the same time, sgp130 is known to be slightly elevated in SLE [44]. Thus, the same type of sIL-6R/sgp130 buffer capacity for IL-6 is formed in SLE. As long as IL-6 levels are moderate, this prevents hepatocyte IL-6 signalling and the ensuing production of CRP. However, if there are higher amounts of IL-6, which can be found in lupus arthritis and serositis [3], as also suggested by the limited data on higher IL-6 levels in this study, both free IL-6 and IL-6/sIL-6R complexes can transmit IL-6 signals in the liver [45], and hepatocytes will produce CRP. Even more so, the more pronounced increase in IL-6 found in bacterial infection will override the ‘buffer capacity’ of sIL-6 receptors and sgp130, and high levels of CRP will be produced. This explains why CRP usually is a marker of severe bacterial infection, but not of active lupus, in patients with SLE.

Patients with RA were primarily included for comparison. As expected, they had a trend towards even higher CRP values and higher IL-6 levels than SLE patients, with a better correlation between these two parameters. RA patients had no significant reduction in either CD126 or CD130 expression on their lymphocytes, but otherwise had findings similar to those in SLE patients. Individual differences were considerable. This may indicate that the same mechanisms may play a role in RA patients, but be reduced in impact by higher IL-6 levels. Indeed, type I IFNs can also be found in subsets of RA patients [46], where their influence would likewise lead to downmodulating CRP, and potentially to underestimating inflammation.

These findings have implications that reach beyond explaining the situation in SLE and potentially RA. This mechanism provides a more general explanation for the usually limited CRP levels even in severe viral disease. Since CRP directly contributes to antibacterial [47], but not antiviral, defence, IFNα-dependent suppression of IL6-dependent CRP induction has resource-saving effects in the context of viral infections. Vice versa, IL-6 trans-signalling may foster anti-viral responses.

This study has limitations. The number of patients was limited, which has implications for the reliability of the statistical comparisons of patient data. It was also not designed to fully analyse the impact of arthritis and serositis on the IL-6–IL-6 receptor system. Moreover, we acknowledge that the step between the *ex vivo* data from patients and the consecutive *in vitro* experiments is not seamless, but includes adaptations, such as a higher and more constant exposure to cytokines. However, the patient numbers appear sufficient for most questions and the consecutive experiments seem reasonably close to the patient situation and appropriate for answering the relevant questions.

Taken together, our data provide evidence that the usually low CRP levels found in SLE are due to shedding of membrane-bound IL-6 receptor and the consecutive increase in soluble IL-6 receptor, induced by IFNα and IL-6 in combination. In addition, our findings demonstrate the importance of disease-associated shifts from classical IL-6 signalling to trans-signalling in a relatively common disease situation. An additional implication is that IL-6 may well play a pathogenic role in SLE, since the relatively low levels of CRP are not due to IL-6 not being elevated, but to the IL-6R shedding induced by IL-6 when in combination with type I IFNs.

## Supplementary Material

keaf281_Supplementary_Data

## Data Availability

Data beyond those directly shown will be shared upon reasonable request.
