# Supplementary material for: Type I interferon limits interleukin-6 signalling in SLE through shedding interleukin-6 receptors
Source: Rheumatology (Oxford). 2025 Jul 9;64(11):5793–802. doi: 10.1093/rheumatology/keaf281 (PMC12596071; doi:10.1093/rheumatology/keaf281)
Supplement: keaf281_Supplementary_Data [file keaf281_supplementary_data.docx]

**Supplementary Figures**

**Figure S1:** In SLE, the correlation between IL-6 and disease activity (by ECLAM) (A) was significant, but not the correlation between disease activity and CRP (B).

**Figure S2:** The correlation between CRP and IL-6 in SLE was lost when only IL-6 values ≤20 pg/mL were included (A), but was maintained in RA (B).

**Figure S3:** Significant negative correlation between sIL-6R and %CD126+lymphocytes in SLE.

**Figure S4:** Decreased ratio of %CD126+lymphocytes over sIL-6R and weak positive correlation between this ratio and the ratio of CRP over IL-6 in SLE.
